# The silence of the neurons: an application to enhance performance and energy efficiency

**DOI:** 10.3389/fnins.2023.1333238

**Published:** 2024-02-28

**Authors:** Moslem Heidarpur, Arash Ahmadi, Majid Ahmadi

**Affiliations:** ^1^Department of Computer and Electrical Engineering, University of Windsor, Windsor, ON, Canada; ^2^Department of Electronics, Carleton University, Ottawa, ON, Canada

**Keywords:** Izhikevich neuron, biological neuron, CORDIC, digital implementation, neuromorphic, spiking neural network, FPGA

## Abstract

**Introduction:**

Simulation of biological neural networks is a computationally intensive task due to the number of neurons, various communication pathways, and non-linear terms in the differential equations of the neuron.

**Method:**

This study proposes an original modification to optimize performance and power consumption in systems, simulating or implementing spiking neural networks. First, the proposed modified models were simulated for validation. Furthermore, digital hardware was designed, and both the original and proposed models were implemented on a Field-Programmable Gate Array (FPGA).

**Results and discussion:**

Moreover, the impact of the proposed modification on performance metrics was studied. The implementation results confirmed that the proposed models are considerably faster and require less energy to generate a spike compared with unmodified neurons.

## 1 Introduction

Inspired by the brain, Spiking Neural Networks (SNNs) represent the third generation of neural networks where neurons communicate through sparse sequences of spikes. In comparison to classic Artificial Neural Networks (ANNs), SNNs have a higher learning and processing speed by considering the timing of events as another variable (Pfeiffer and Pfeil, 2018). Furthermore, SNNs are orders of magnitude more energy-efficient due to a low spiking rate and activity. Such networks are also useful for studying biological neural networks and brain diseases (Pastur-Romay et al., 2016). New learning algorithms for spiking neural network are also evolving (Yang and Chen, 2023a,b; Yang et al., 2023b). Yang et al. (2024) proposes a neuromorphic architecture designed for online learning through dendrites to improve the efficiency of spike-driven learning and enhance the performance of processing spatiotemporal patterns. A novel fault-tolerant address event representation approach is proposed the study by Yang et al. (2023a) for the spike information routing to improve the efficiency and reliability of smart traffic navigation.

Several computational models have been proposed to mimic biological neurons (Hodgkin and Huxley, 1990; Gerstner and Kistler, 2002; Izhikevich, 2003; Brette, 2005). In these models, there is often a trade-off between being concise and computationally efficient or being biologically plausible and complex.

Simulation of SNNs is computationally complex due to the number of neurons, various communication pathways, and non-linear terms in the differential equations describing these models. As implementation platforms, researchers have used PCs (NeMo, 2024; The Brian Simulator, 2024), supercomputers (EPFL, 2024), analog (Schemmel et al., 2010; Covi et al., 2015), digital (Merolla et al., 2011; Akopyan et al., 2015; Barchi et al., 2019), mixed analog/digital VLSI neuromorphic hardware (Benjamin et al., 2014; Wang et al., 2014; Neckar et al., 2019), and FPGAs (Neil and Liu, 2014; Liu et al., 2018; Heidarpur et al., 2019), to simulate and realize SNNs. Each platform comes with its own set of strengths and weaknesses. Nevertheless, in all aforementioned platforms, enhancing speed and minimizing energy consumption are very important.

An important contributing factor to the computational complexity of neuron models is calculating non-linear terms in their differential equations. In view of this, researchers have proposed a variety of approaches to speed up systems simulating or implementing spiking neurons where, in general, accuracy is exchanged for performance. Such methods include Piece-Wise Linear (PWL) approximation (Yamashita and Torikai, 2014; Heidarpur et al., 2017), Coordinate Rotation Digital Computer (CORDIC) (Heidarpour et al., 2016; Elnabawy et al., 2018), an asynchronous cellular automaton used in Matsubara and Torikai (2013), a nonlinear function evaluation technique (Jokar et al., 2019), bit-serial reduced-range multipliers (Karim et al., 2017; Kueh and Kazmierski, 2017), and a novel rotate-and-fire neuron (Hishiki and Torikai, 2011), among others.

Power dissipation and density are another important concern and one of the major challenges that need to be resolved for the massive large-scale implementation of neuromorphic systems. Total power dissipation is the sum of two components: static and dynamic power dissipation. Dynamic power is associated with activity and switching events in the core or I/O of the device (Rabaey et al., 2003). Power optimization could be at the circuit, logic, architectural, or system levels (Devadas and Malik, 1995). Various methods, mostly at the circuit and logic levels, are suggested to reduce power consumption of spiking neural networks (Lee et al., 2004; Indiveri et al., 2006; Tao and Rusu, 2015; Kohno and Aihara, 2016). Analysis of static power, which is independent of circuit activity and primarily from transistor leakage, is out of the scope of this study.

For computer simulation and digital hardware implementation, the differential equations describing spiking neurons are discretized and numerically solved by evaluating them at every time step, even when a neuron is silent. In this study, through the observation of both the input current and the derivative of the membrane potential, we discovered that the amount of computation could be reduced when a neuron is silent or spikes at a slow rate. Based on this insight, we proposed a modification to avoid computing certain terms of the differential equations in the spiking neuron model. During a fast spiking state, the neuron can switch back to the full calculation of Ordinary Differential Equations (ODEs). The proposed technique has the potential to save energy and time, making it particularly valuable when implementing large networks. In the case of software implementation, the proposed improvement over spiking neuron can reduce the total number of times that ODEs are evaluated and therefore reduce the total number of computation required for simulating neuron. In this study, software simulations were performed to measure the improved efficiency resulted from applying proposed method. Additionally, in the case of hardware implementation, the proposed method reduces the total number of computation required and also saves the energy consumption, which is the most useful if devices are operated on battery. The main focus of the paper remains the advantages of proposed method for hardware implementation.

As a case study, the Izhikevich neuron model (Izhikevich, 2003) was modified using the proposed technique and further simulated for validation. Additionally, a network consisting of both the original and modified models was developed, trained, and implemented on hardware to study the effects of the proposed modification on both accuracy and performance.

The rest of the study is organized as follows: Section 2 reviews the Izhikevich neuron and presents the proposed method to save computations in neuronal differential equations. Section 3 investigates the validity of the proposed models through error analysis and studies their impacts on computer simulation performance. Section 4 discusses the FPGA implementation procedure and how the proposed modification optimizes energy consumption and speed of hardware. Finally, Section 5 concludes the study.

## 2 Proposed duplex neuron

This section presents a modification to optimize performance and power consumption in systems simulating or implementing spiking neural networks.

### 2.1 Background

**Figure 1:**
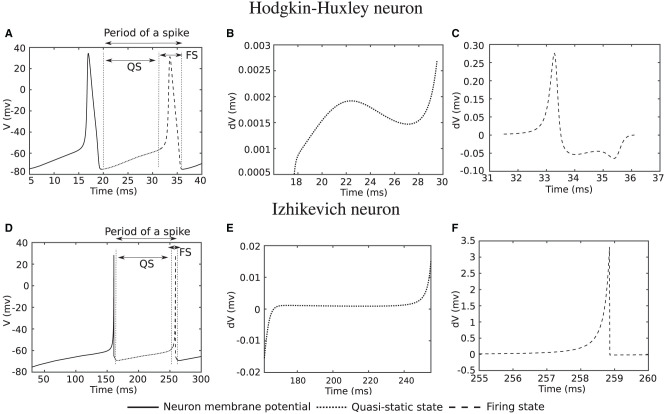
Computer simulation of Hodgkin-Huxley and Izhikevich neuron model for a constant input current. **(A, D)** Action potential of neurons. **(B, E)** Derivative of action potential during Quasi-Static (QS) state. **(C, F)** Derivative of action potential during firing state (FS).

Researchers have presented various models to simulate and study the behavior of biological neurons. These models are formulated as coupled differential equations that need continuous evaluation over time, which typically involves numerical methods since spiking neuron models do not have analytical solutions. Furthermore, bifurcation analysis helps to study qualitative changes in the dynamics of neuron as a function of certain parameters, such as synaptic strengths, time constants, or external inputs (Izhikevich, 2007). One of the bifurcation parameters, determining the states of a neuron, transitioning from silent to firing, and influencing its rate, is the input current. In Figure 1, the simulation of two well-known spiking neuron models, Hodgkin–Huxley (HH) (Hodgkin and Huxley, 1990) and Izhikevich (Izhikevich, 2003), is depicted for a constant input current. The Hodgkin–Huxley (HH) neuron model is a mathematical model that describes the generation and propagation of action potentials, or spikes, in biological neurons, whereas the Izhikevich neuron model is a simplified model that aims to capture essential features of neuronal dynamics while minimizing computational complexity

In Figure 1A, the membrane potential (*V*) of a Hodgkin–Huxley neuron is demonstrated when the input current (or its integral over time) is sufficiently high in transition of the neuron from silence to firing. The derivative of the membrane potential over the dotted line in Figure 1A is shown in Figure 1B. In this state, which we further refer to as the quasi-static (QS) state, the derivative tends to remain almost constant with very small changes. The derivative of the membrane potential over the dashed line in Figure 1A is depicted in Figure 1C. The value of the derivative for this state [hereafter referred to as the Firing State (FS)] is considerably larger compared with that over the dotted line. The same scenario also applies to the Izhikevich neuron, with the corresponding simulations shown in Figures 1D–F. What is interesting is that for the Hodgkin–Huxley neuron, the quasi-static state makes up ~75% of the spiking period, which is a significant portion. This percentage for the Izhikevich neuron is even higher, roughly ~95%. The Izhikevich neuron is in the firing state for only ~5% of a spike period.

**Figure 2:**
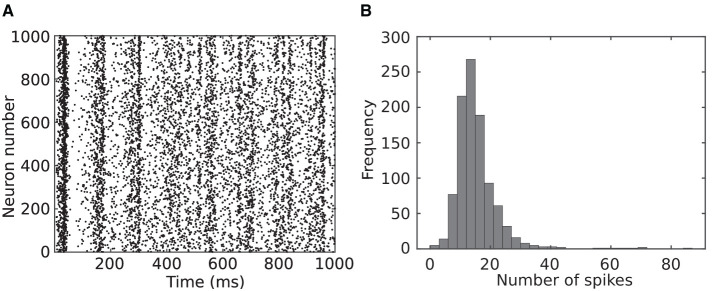
One thousand Izhikevich neurons were randomly coupled and simulated for 1,000 ms following the method in the study by Izhikevich (2003). **(A)** Spike raster diagram where each dot represents a specific neuron spiking at a specific time. **(B)** Distribution of number of spikes for neurons in the network.

The previous paragraph studied neuron behaviors while spiking consistently. To further investigate the behaviors of spiking neuron models, 1,000 Izhikevich neurons were randomly coupled and simulated according to the method utilized in reference (Izhikevich, 2003). Figure 2A shows the spike raster diagram for this network. Raster diagram provides a visual representation of the timing of action potentials or spikes across multiple neurons over time. Each vertical line in this diagram represents the occurrence of a spike, and the horizontal axis corresponds to time. Raster diagrams provide an intuitive and informative way to visualize the precise timing of spikes from individual neurons and can be used to study the patterns of spike synchrony or specific temporal relationships. Coherent activity could be observed as vertical columns of dots, which is similar to the temporal synchrony (alpha and gamma band rhythms) of neurons observed in biology (Vaidya and Johnston, 2013).

Distribution of number of the spikes for the neurons in this network is presented in Figure 2B. As this figure indicates, most of the neurons (almost 90 %) spike between 6 and 24 times during 1 s of simulation and are silent rest of the time. This indicates the potential of the proposed modification to save computation in a spiking neural network.

In the following sections, Izhikevich neuron was considered as case study, where the proposed method was applied to a single neuron and also an spiking neural network to evaluate performance.

### 2.2 The duplex neuron

Izhikevich neuron is a two-dimensional model consists of two coupled ODEs as follows:


(1) $$\begin{array}{l} \frac{dv}{dt}=0.04v^{2}+5v+140-u+I \end{array}$$

(2) $$\begin{array}{l} \frac{du}{dt}=a\left(bv-u\right) \end{array}$$

along with an after-spike reset equation as follows:


(3) $$\begin{array}{l} if\;v>30mv\;then\;\{\begin{array}{c} v\to v_{r}\\ u\to w_{r}=u+d. \end{array} \end{array}$$

where *v* represents membrane potential, *u* is recovery variable, and *I* stands for injected current to the neuron. Other dimensionless parameters are as follows:

*a* : Time scale of the recovery variable
*b* : Sensitivity of the *u* to *v*
*c* : After-spike reset value of *v*
*d* : After-spike reset value of *u*

By regulating these parameters, the Izhikevich model can mimic different neuronal behaviors observed in biological neurons. To simulate this model, Equations 1, 2 were discretized as follows:


(4) $$\begin{array}{l} \begin{array}{c} v\left[n+1\right]=\left(0.04v{\left[n\right]}^{2}+5v\left[n\right]+140-u\left[n\right]+I\left[n\right]\right)dt+v\left[n\right]\\ u\left[n+1\right]=a\left(bv\left[n\right]-u\left[n\right]\right)dt+u\left[u\right] \end{array} \end{array}$$

and numerically solved using the Euler method.

**Figure 3:**
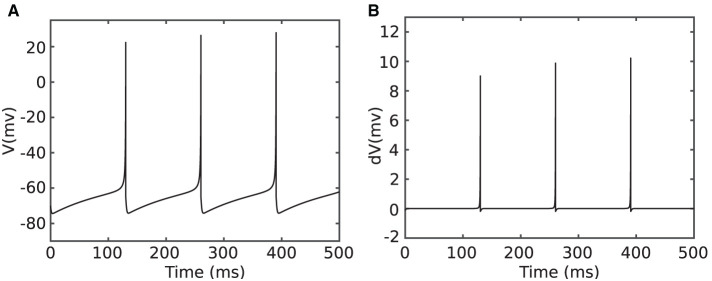
Simulation of a tonic spiking Izhikevich neuron which is stimulated with a constant current (Equation 4). **(A)** Membrane potential *v*[*n*] and **(B)** membrane potential derivative *v*[*n* + 1] − *v*[*n*].

Figure 3A shows simulation of a tonic spiking Izhikevich neuron stimulated with a constant input current. The neuron parameters for this simulation are: *a* = 0.02, *b* = 0.2, *I* = 4 mA, and *dt* = 1/32 ms. Figure 3B plots the derivative of the membrane potential (*v*[*n* + 1] − *v*[*n*]). As shown in this figure, the derivative is approximately constant and close to zero (quasi-static state), except when the neuron membrane goes up for a spike (firing state). The small value of the derivative for the quasi-static state implies a small change in action potential when the neuron is in the quasi-static state or when it is silent. To benefit from this property, Equation 4 was modified as follows:

When the neuron is silent or in the quasi-static state, α and β do not change significantly, and the last values of these parameters can be used to evaluate neuron ODEs instead of calculating them every time. The modified model is hereafter referred to as the Duplex (DX) neuron. The term 5*v*[*n*] was excluded from α since the coefficient is relatively larger than other parameters, considering that it constant affects neuron behaviors. The parameter δ is the threshold value that determines whether it is necessary to update α and β or if the last updated value is still valid. A larger value of delta results in greater computational savings but induces higher error and vice versa. Therefore, determining the proper δ is a compromise between accuracy and performance. In the following, we investigate this trade-off.

## 3 Computer simulation

This section investigates the impacts of proposed modification on error and performance of a single and a network of Izhikevich neurons.

### 3.1 Error analysis

The objective of this section is to investigate whether the neurons with the proposed modification still have a valid behavior, which is similar to unmodified neuron.

#### 3.1.1 Qualitative comparison

**Figure 4:**
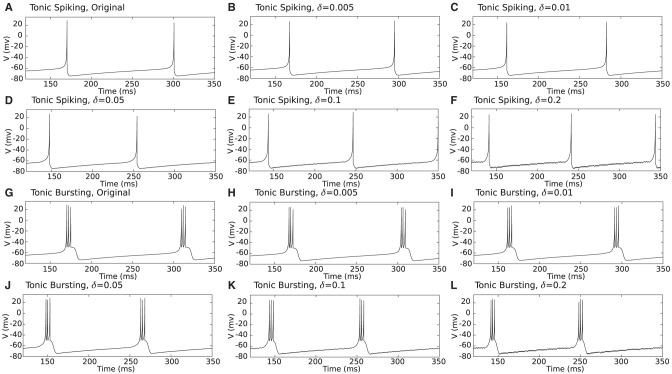
**(A–L)** Computer simulation of a tonic spiking/bursting Izhikevich and proposed duplex Izhikevich neuron for different values of δ. As it is evident in this figure, even for large values of δ such as 0.2 mv, besides small oscillations, waveform of the membrane potentials (*v*) are close to those of the unmodified model.

First, for qualitative comparison, proposed duplex Izhikevich neurons were simulated for different values of the δ, as shown in Figure 4. As shown in this figure, even for greater values of delta such as δ = 0.2 mv, besides small oscillations, the membrane potential waveform of the proposed duplex neuron is close to that of the model which constantly evaluates the entire differential equation. Nevertheless, proposed modification evidently results in some discrepancies in timing of spikes.

#### 3.1.2 Quantitative comparison

In the following, the deviations in the previous section were quantified by calculating the following errors:

Normalized root mean square deviation (NRMSD): This error was calculated to measure the deviation induced by different values of δ to the shape of the membrane potential waveform. Higher resemblance between the form of the spikes results in a lower value for this error. This error is calculated as follows:


(5) $$\begin{array}{l} NRMSD=\frac{\sqrt{\left(\overset{n}{\underset{i=1}{\sum }}{\left(v_{Un}\left(n\right)-v_{DX}\left(n\right)\right)}^{2}\right)/\left(n\right)}}{v_{Unmax}-v_{Unmin}}\times 100 \end{array}$$

where *V*_*DX*_ and *V*_*Un*_ are waveforms of the duplex and unmodified Izhikevich neurons. *v*_*Unmax*_ and *v*_*Unmin*_ are the minimum and maximum of membrane potential (*v*) between *v*[1] and *v*[*n*], where *n* is the total number of points that this error is evaluated. Timing error (TE): This error measures the difference in the time interval between two consecutive spikes and is calculated as follows:


(6) $$\begin{array}{l} TE=\left|\frac{\Delta ts_{Un}-\Delta ts_{DX}}{\Delta ts_{Un}}\right|\times 100 \end{array}$$

where Δ*ts*_*Un*_ and Δ*ts*_*DX*_ are spike periods of the unmodified and proposed duplex Izhikevich neuron.

These errors were calculated for a tonic spiking Izhikevich neuron which was simulated with an input current of 4 mA and a time step of 1/32 ms and are presented in Table 1. As data in this table indicate, these errors are higher for larger values of δ. However, the increase rate almost levels out for δ > 0.01 mv.

**Table 1:** Errors, computer saving percentage, and speed up percentage for computer simulation of proposed duplex neuron on the basis of δ (mv).

**δ (mv)**	**TE% (Eq. 6)**	**NRMSD% (Eq. 5)**	**CSP% (Eq. 7)**	**Speed up % (Eq. 8)**
0.001	0.04	0.11	00.3	2.52
0.005	1.87	0.12	58.2	10.47
0.010	6.67	0.31	85.3	16.78
0.050	18.25	1.12	91.8	18.19
0.100	20.74	1.51	92.3	18.31
0.200	23.22	1.79	93.7	18.53

The neuron was stimulated with a constant input current of 4 mA.

#### 3.1.3 Network behavior

**Figure 5:**
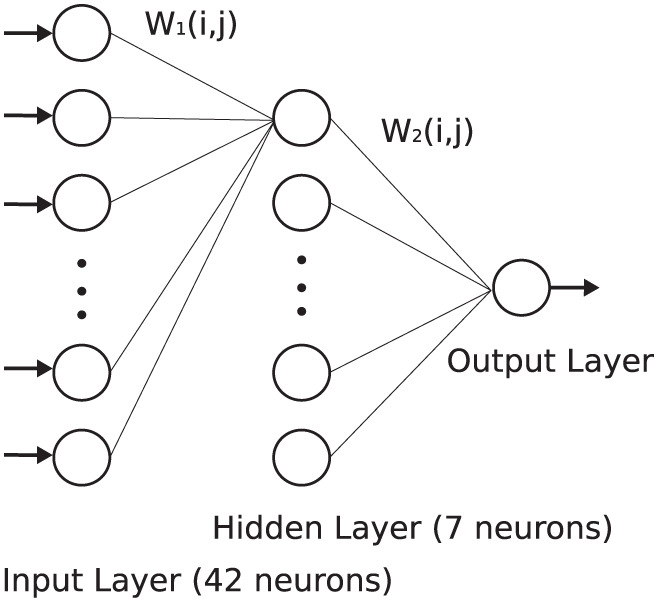
To study behavior of the modified model, a three-layer network of the Izhikevich neurons was developed and trained according to the method presented in references Christophe et al. (2015) and Farsa et al. (2019).

To study the behavior of the proposed duplex model in an application, a basic three-layer network of Izhikevich neurons was constructed, as shown in Figure 5. The network comprises 42 neurons in the first layer, seven neurons in the second layer, and, finally, one neuron in the output layer. This network was trained using the Spike Time Dependent Plasticity (STDP) rule to recognize two patterns of *E* and *H*, following the method used in references Christophe et al. (2015) and Farsa et al. (2019). First, weights were initialized randomly. Furthermore, the network was stimulated with the pattern *E* during the training phase. In the next phase, both patterns of *E* and *H* were applied randomly to the input of the network to test its validity. For more details, please refer to references Christophe et al. (2015) and Farsa et al. (2019).

**Figure 6:**
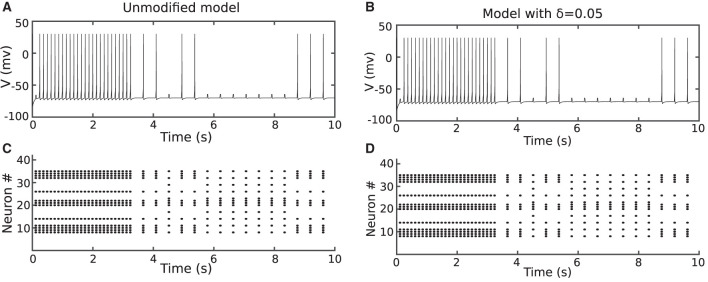
The network in Figure 6 was trained using the method described in Christophe et al. (2015) and Farsa et al. (2019). **(A, B)** Shows the output neuron membrane potential and network raster diagram during testing phase for unmodified model. **(C, D)** Shows the same diagrams for the proposed duplex Izhikevich neuron. The objective of this figure is to demonstrate that the network behavior of the modified model is similar to that of the original model.

To evaluate the behavior of the proposed models, the network was trained with both duplex and original Izhikevich neurons. Figures 6A, C show the membrane potential of the output neuron for the original and the modified model with δ = 0.05 mV. The corresponding raster diagram for the networks during the training and testing phases is shown in Figures 6B, D. In the previous section, the input current of the neuron was presumed to be constant. On the other hand, in a practical application or biology, the input current is not steady and may abruptly change. The results of this test confirm that the behavior of the proposed duplex neuron remains close to the original model even with interrupted input currents.

### 3.2 Performance analysis

This section investigates the impacts of proposed modification on computer simulation performance.

#### 3.2.1 Single neuron

In the proposed duplex neuron, there is no need to compute α and β terms (Algorithm 1) constantly. The percentage of iterations that these terms are computed in the duplex neuron to the total number of iterations determines the performance improvement. Therefore, Computation Saving Percentage (CSP) was defined and formulated as follows:


(7) $$\begin{array}{c} CSP=\frac{Number\;of\;iterations\;\alpha \;and\;\beta \;are\;not\;computed}{Total\;number\;of\;iterations}\;\times 100 \end{array}$$

**Algorithm 1:** Proposed duplex neuron.

1: if |*v*[*n* + 1] − *v*[*n*]| > δ **then**
2: α ← 0.04*v*[*n*]^2^ + 140 − *u*[*n*]
3: β ← *a*(*bv*[*n*] − *u*[*u*])
4: else
5: *do nothing*
6: end **if**
7: *v*[*n* + 1] = (α + 5*v*[*n*] + *I*[*n*])*dt* + *v*[*n*]
8: *u*[*n* + 1] = (β)*dt* + *u*[*u*]

CSP for different values of δ is presented in Table 1. According to the results in this table, for δ > 0.01, more than 90% of the time, there is no need to constantly calculate the square function in Izhikevich neuron ODEs. To study performance improvement, original and duplex neurons with different values of δ were simulated for 20 s in MATLAB software, and total execution times were measured to calculate the Speed Up Percentage (SUP) as follows:


(8) $$\begin{array}{c} Speed\;up=\frac{T_{DX}-T_{Un}}{T_{Un}}\times 100 \end{array}$$

where *T*_*DX*_ and *T*_*Un*_ are execution time of proposed duplex and unmodified Izhikevich model, respectively. Speed up percentage for various values of δ is presented in Table 1. As data suggest, speed up is higher for larger values of δ. Nevertheless, following the pattern of errors, speed up also levels out after δ > 0.01 mv.

#### 3.2.2 Neurons in a network

**Figure 7:**
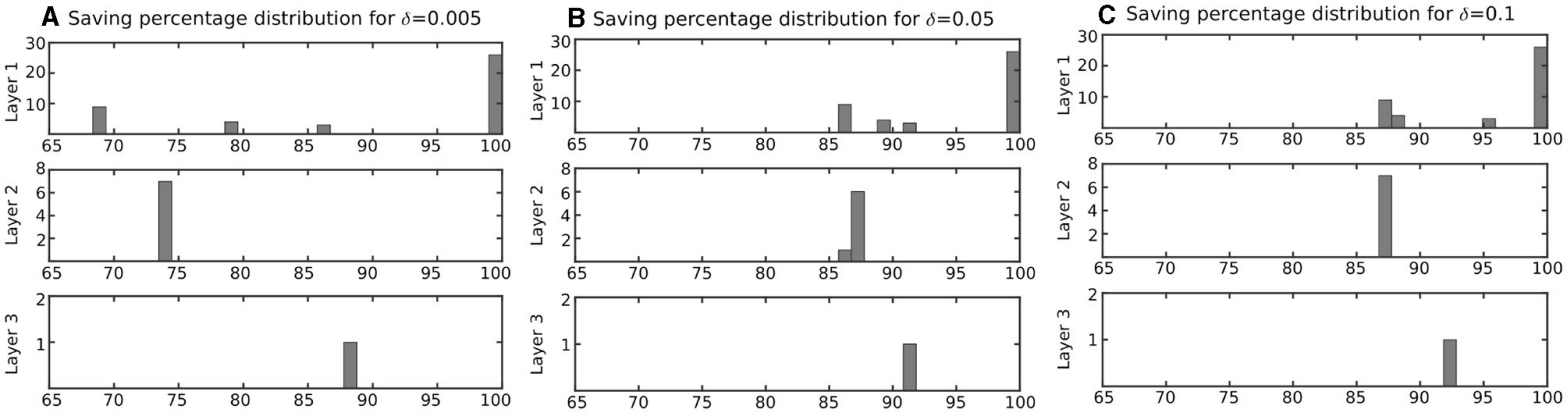
Distribution of computation saving percentage for the neurons in network of Figure 5 based on layer. Computation saving percentages were measured during a testing phase. **(A)** Neurons with δ = 0.005 mv, **(B)** Neurons with δ = 0.05 mv, and **(C)** Neurons with δ = 0.1 mv. This figure indicates that computation-saving percentage of the proposed modified model is even higher in a network, where the neuron is not active all the time in comparison with a single neuron that is stimulated with a constant input current (results in Table 1).

The neuron in the previous section was stimulated with a constant current to spike continuously. However, as discussed before, this is not the case in a network of spiking neurons. Therefore, it is expected that the proposed duplex neuron features even better performance improvement in a network. To investigate this, the computation saving percentage for each neuron in the network, as shown in Figure 5, was calculated during the testing phase. The results for neurons in each layer are presented in Figure 7 on the basis of δ.

Figure 7 column (A) shows the computation saving percentage for duplex neurons with δ = 0.005 mV. Such CSPs are much higher than those calculated for the neurons with a constant input current (Table 1). Indeed, even for the neurons with relatively small δ, which have very low error, the computation saving percentage is considerable in a practical application.

#### 3.2.3 Input current

Higher spike rate results in shorter QS period during a spike. This, in turn, leads to lower computation saving percentage and, as a consequence, lower performance improvement.

**Figure 8:**
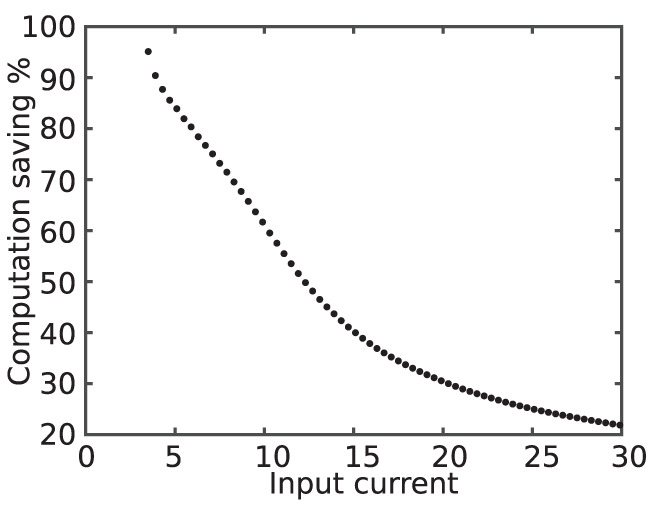
Computation saving percentage as a function of the input current. The higher value of the input current results in higher activity, smaller QS state period, and, consequently, declining computation saving percentage.

Figure 8 shows the computation saving percentage as a function of the input current. As it is evident in this figure, computation saving percentage is lower for larger input currents.

In contrary, the quasi-static state makes up the majority of the time for the neurons that rarely fire. Therefore, for silent neurons, the proposed modification saves more computation. In addition to improving execution time, proposed modification also saves energy by decreasing switching activity during simulation.

## 4 Hardware implementation

This section discusses hardware design and implementation of the proposed duplex neuron models on FPGA and interpretation of results.

### 4.1 Hardware design

**Figure 9:**
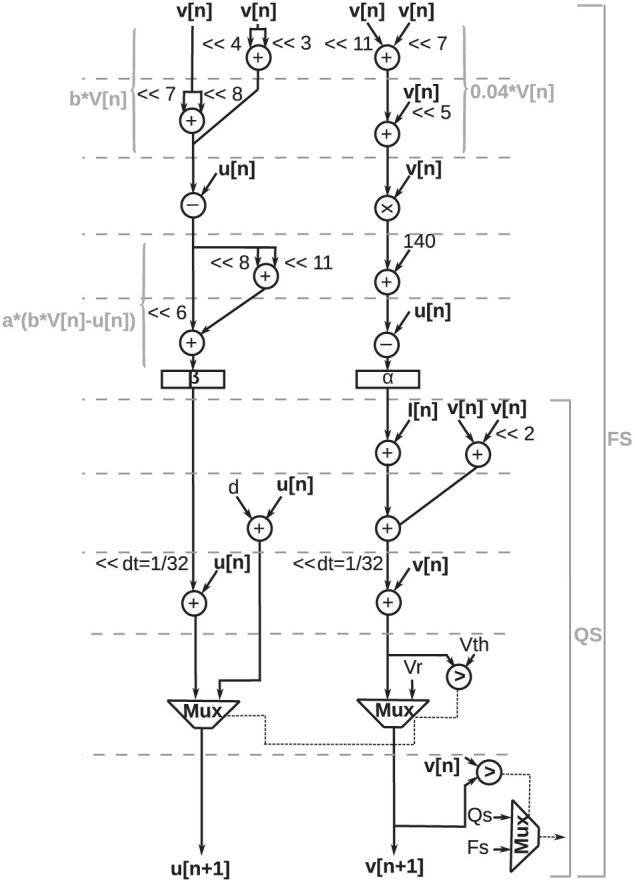
Data flow graph for digital implementation of proposed duplex Izhikevich neuron. A comparator and multiplexer determine whether a neuron is in the quasi-static state (QS bracket) or the firing state (FS bracket) by comparing the updated value of action potential (*v*[*n* + 1]) with its previous value (*v*[*n*]). As shown in this figure, if neuron is in quasi-static sate, critical path is shorter, and there is no need to compute square term which is slowest and most complex operation.

The Data Flow Graph (DFG) for digital implementation of the discretized Izhikevich equations (Equation 4) and reset equation (Equation 3) is shown in Figure 9.

In this illustration, addition, subtraction, and multiplication operations are represented as circles. A comparator and multiplexer determine whether the neuron is in the quasi-static state or the firing state by comparing the updated value of the action potential (*v*[*n* + 1]*v*[*n* + 1]) with its previous value (*v*[*n*]*v*[*n*]). As shown in this figure, if the neuron is in the quasi-static state, the critical path is shorter, and there is no need to compute the square term, which is the slowest and most complex operation.

Values of α and β are stored in two registers (shown as rectangles). The operation units are scheduled in a way that no more than two adders are needed at any time. Arithmetic shift operations are denoted by “≪” symbol, where “≪2” indicates that data shifted twice to the left. Multiplication with constants is performed using add and shift operations. For instance, 0.04 × *v*[*n*] was calculated as follows:


(9) $$\begin{array}{c} \begin{array}{c} 0.04\times v\left[n\right]\approx \left(0.0396\right)v\left[n\right]=\left(2^{-11}+2^{-7}+2^{-5}\right)v\left[n\right]\\ =\left(v\left[n\right]<<11\right)+\left(v\left[n\right]<<7\right)+\left(v\left[n\right]<<5\right) \end{array} \end{array}$$

Such approximation causes a small error; however, it considerably improves the performance of the design. A CORDIC algorithm, as presented in the study by Heidarpur et al. (2019), was used to perform the square function in neuron ODEs. Fixed-point arithmetic was utilized since fixed-point units are considerably cheaper and faster compared with floating-point units. Furthermore, the word length of the design was determined considering the number of integer bits to represent variables in their domain and the number of fraction bits for the minimum required precision. Additional bits were also added to avoid overflow or underflow. Taking these requirements into consideration, a 30-bit word length was specified, comprised of a 14-bit fraction and a 16-bit integer part.

### 4.2 Hardware implementation

To implement the design on FPGA, the architecture, as shown in Figure 9, was modeled in Very High-speed integrated circuit Hardware Description Language (VHDL). The design was verified by simulating and testing using ModelSim software. Subsequently, the HDL description was synthesized and configured for FPGA implementation using Xilinx Integrated Synthesis Environment (ISE) software tool.

#### 4.2.1 A single neuron

**Figure 10:**
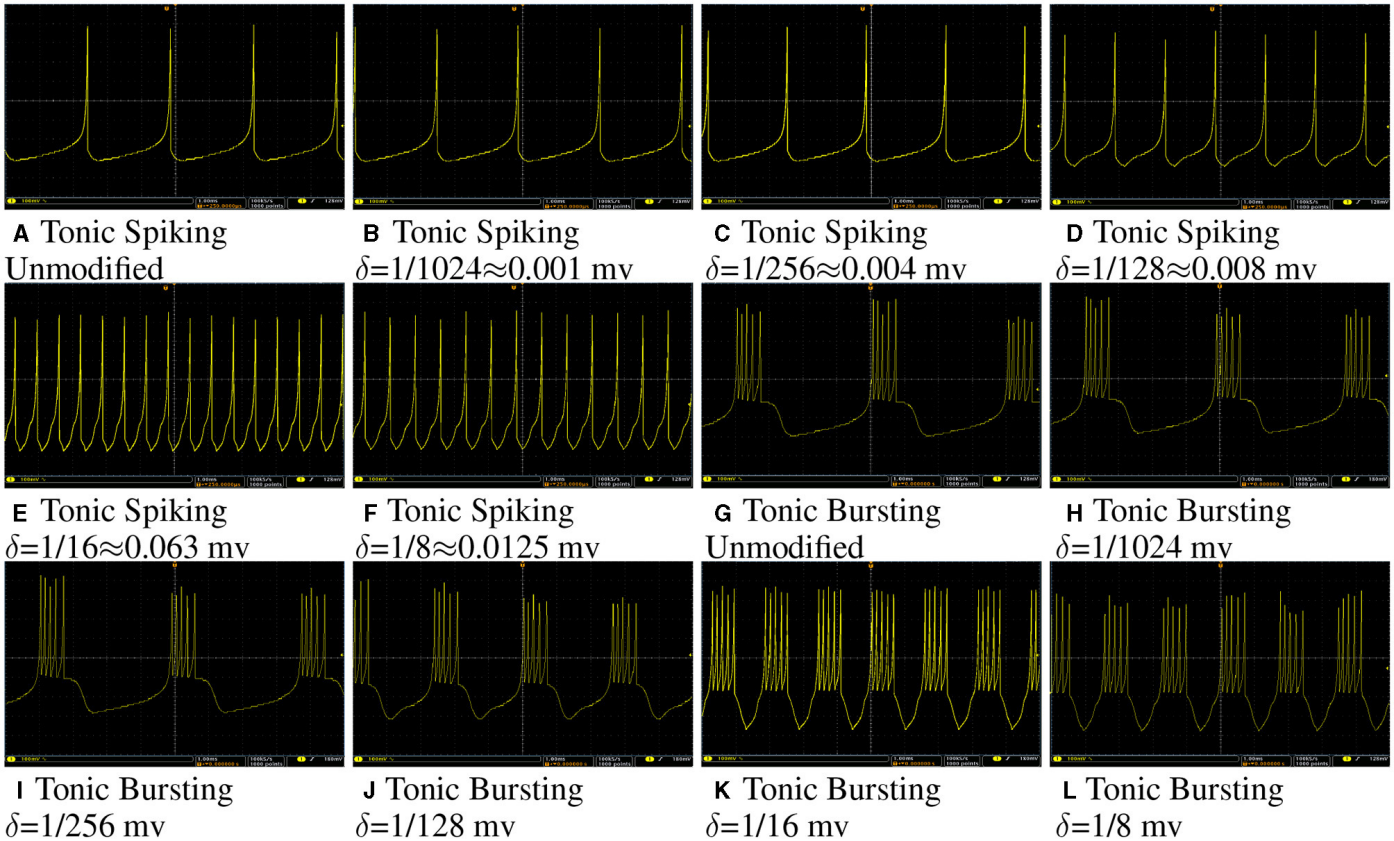
**(A–L)** Oscilloscope photos of FPGA implementation of a tonic spiking/bursting Izhikivech and duplex proposed neurons with different values of δ. The input current for all of these case is 16 mA. The oscilloscope time scale and volt scale for all of figures are 1 ms and 100 mv, respectively. This figure demonstrates that the proposed hardware for the modified Izhikevch neuron has the same behavior as the hardware of the original neuron.

Figure 10 shows membrane potential of a tonic spiking/bursting Izhikivech and a duplex proposed neuron for different values of δ on Spartan 6 XC6LX75 FPGA. The data were converted to analog using a 12 bit Digital to Analog Converter (DAC).

As this figure demonstrates, even for large values of δ, such as 0.128 mv, the duplex model still follows the original model. However, by increasing δ, one can observe some differences in the shapes of spikes. The input current for all neurons is the same, but spike rate for neurons with larger δ are higher since they have higher throughput.

**Figure 11:**
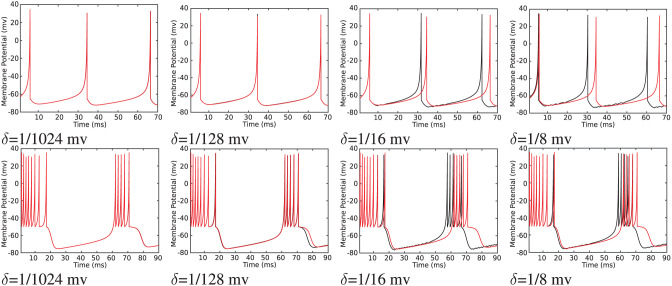
The on-FPGA data were transferred to PC through UART port, recovered and plotted for the Izhikevich neuron (red lines) and proposed duplex neuron with different values of δ (black lines). Duplex neurons with relatively large δ, such as 1/8 mv, still follow the unmodified model except for small changes in the shapes of spikes.

To transfer data from FPGA to PC for further analysis, a Universal Asynchronous Receiver-Transmitter (UART) module was developed and implemented on FPGA as described in the reference Heidarpur et al. (2019). Transferred data are presented in Figure 11. As it is evident in this figure, the proposed and unmodified model has a similar membrane potential waveform apart from some deviations in the shape of spikes. These implementation results are in agreement with computer simulation results in Computer Simulation Section, as presented in Figure 4 and Table 1.

#### 4.2.2 A network of neurons

**Figure 12:**
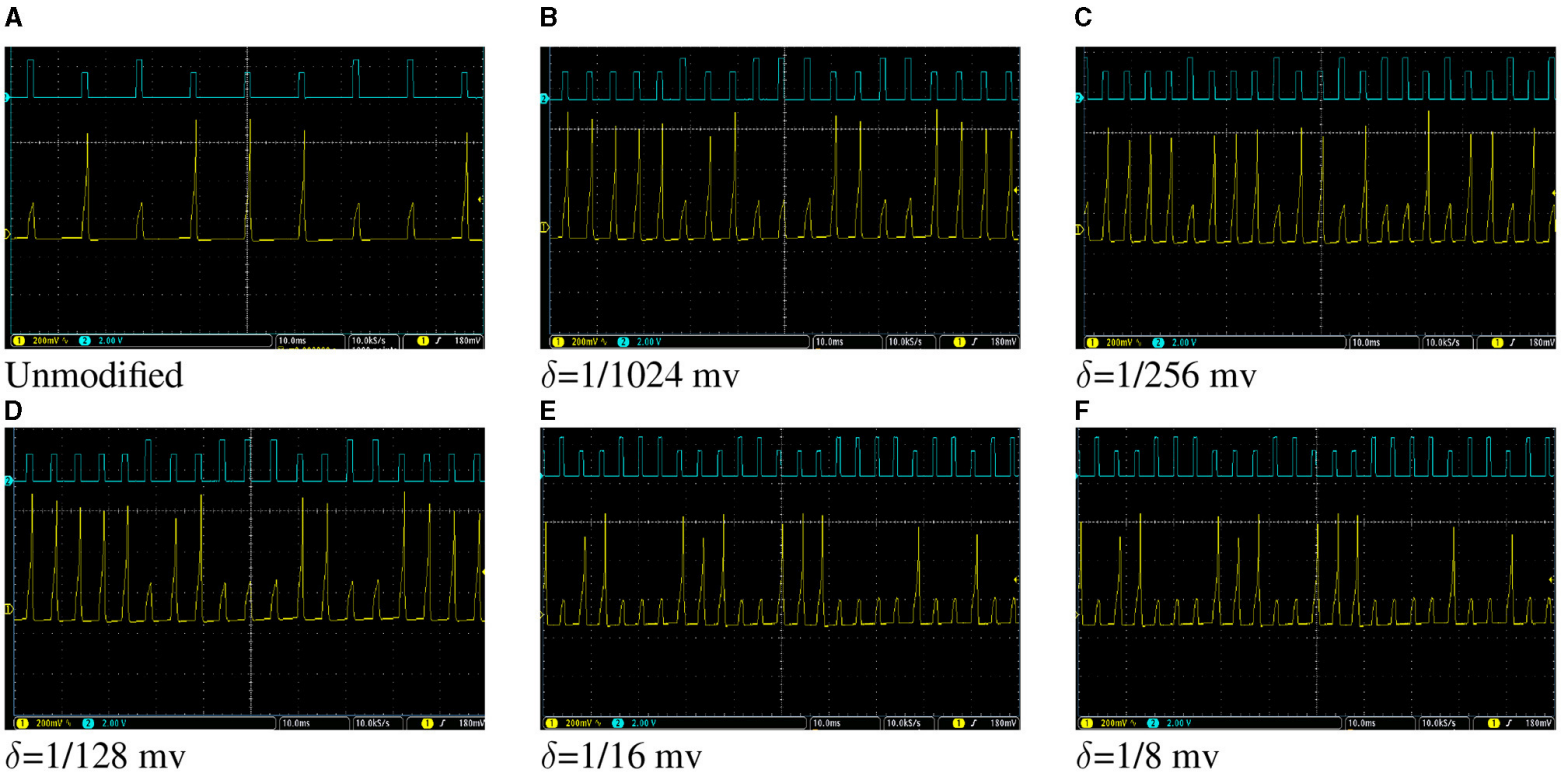
**(A–F)** Oscilloscope photos of FPGA implementation of the network of Izhikevich neurons in Figure 5 for a very basic pattern recognition application. The pulses with higher amplitude indicate where the letter “E” is applied to the network, and pulses with lower amplitudes stand for the letter “H.” The output neuron spikes when “E” is applied and is silent otherwise. The oscilloscope time scale for all figures is 10 ms. The oscilloscope volt scale for figures showing the membrane potential of the output neuron is 200 mV and for figures showing patterns is 2V. The objective of this figure is to demonstrate that the hardware designed for the modified model has the same behavior in a network as unmodified Izhikevich neurons, despite the fact that the network of the modified models is computationally cheaper and consumes less energy because of lower switching activity.

The network, as shown in Figure 5, was described in VHDL and implemented on Spartan 6 XC6LX75 FPGA. Figure 12 shows oscilloscope photos of membrane potential of output neuron of the network while tests are applied to the network. Figure 12A shows the result for the network of the unmodified Izhikevich neurons where the output neuron spikes for “E” and is silent for “H.” Thereafter, the same network with proposed duplex neurons was implemented on FPGA. While implementing modified models with different values of δ, we faced two unexpected challenges.

First, in the unmodified Izhikevich neuron, all neurons generate new output after a certain number of the clock cycles and have the same throughput. However, for the duplex neuron, this number could be different for each neuron depending on its input weights. Transferring data from one layer to the next layer requires that all neurons action potentials of that layer to be updated. Therefore, the slowest neuron determines the critical path, and faster neurons must wait in idle state for others to finish.

Second, the proposed modification results in discrepancy in timing of the spikes since the duplex neurons are faster than unmodified neurons. On the other hand, SNNs are primarily based on timing. Because of that, the network, initially, did not function properly with the weights calculated based on the original neuron and was not able to recognize all instances of input patterns. For larger values of δ, where the speed up was more dramatic, overall network failure rate was higher. To resolve this issue, the weights were recalculated by applying STDP rule to network of duplex neurons. Figure 12 shows oscilloscope photos of FPGA implementation of the trained network, where two test patterns are applied to network. It could be observed in this figure that the on-FPGA network of duplex neurons, even for large values of δ, has the same behavior as the network of original Izhikevich neurons.

### 4.3 Results and discussion

The objective of the proposed modification is to reduce the number of the clock cycles required to generate an output. This contributes to increasing speed and reducing power consumption of the circuit. Table 2 compares resource utilization and operation frequency for FPGA implementation of the original and duplex modified Izhikevich neuron. The proposed modification does not result in a notable increase in resources that are needed to implement the neuron hardware on FPGA. Furthermore, resources and frequency are comparable to similar studies. Some studies reported low resources but they use extensive approximations and are not very accurate replicators of Izhikevich neuron. Table 3 shows on-FPGA dynamic power consumption reported by Xilinx Power Estimator (XPE), and the number of the clock cycles and time is required to generate a spike.

**Table 2:** Comparison between resource utilization and frequency of the proposed method and previously published studies.

**References**	**Slice registers**	**Slice LUT's**	**Frequency**	**DSPs**	**Device**
Soleimani et al. (2012)	493	617	241.9	0	Virtex-II Pro XC2VP30
Haghiri et al. (2018)	490	459	240	0	Virtex-II Pro
Yang et al. (2020)	130	119	291.8	-	ZCU102
Jokar et al. (2019)	195	198	285	0	Virtex-6
Grassia et al. (2014)	646	1,048	105	22	Virtex-5 XC5VLX50
Heidarpour et al. (2016)	829	1,221	134.3	0	Spartan-6 XC6SLX9
Shimada and Torikai (2015)	357	1,776	–	–	Zync-7000 XC7Z020
Original Izhikevich	270	469	212.8	0	Spartan-6 XC6SLX75
Duplex Izhikevich	297	532	171.9	0	Spartan-6 XC6SLX75

Objective of this study is not to reduce resource utilization or increase the frequency of design but rather to skip unnecessary computations to save power and reduce latency of the neuron.

**Table 3:** On FPGA dynamic power, total number of clock cycles and total time required for unmodified and proposed DX Izhikevich neurons with different values of δ (mv) to generate a spike.

	**Unmodified**	**δ=1/1,024**	**δ=1/256**	**δ=1/128**	**δ=1/16**	**δ=1/8**
Dynamic power (mW)	11	6	6	6	6	6
Number of clocks	122,400	120,788	114,704	73,780	37,312	32,200
Total time (ms)	2.45	2.42	2.30	1.47	0.74	0.65

#### 4.3.1 Speed

**Figure 13:**
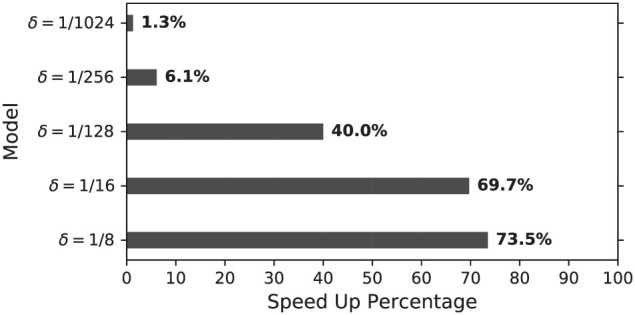
Speed up percentage for on-FPGA duplex models with different values of δ (mv) (Algorithm 1). Speed up percentage is considerably higher for larger values of δ while the neuron behavior does not change notably as demonstrated before.

As data in Table 3 indicate, proposed modification, especially for larger values of *delta*, considerably decreases total computation time per spike. Furthermore, speed up percentage was calculated according to Equation 8 and compared as follows


(10) $$\begin{array}{c} Speed\;up=\frac{T_{Un}-T_{DX}}{T_{Un}}\times 100 \end{array}$$

where *T*_*Un*_ and *T*_*DX*_ are total computation time per spike for unmodified and duplex Izhikevich neuron. This percentage was calculated and compared for different values of *delta*, as shown in Figure 13. As this figure indicates, models with δ = 1/8 mv and 1/16 mv require almost 70% less computation time to generate a spike.

#### 4.3.2 Power dissipation

Reported by XPE, original Izhikevich model dynamic power on FPGA is almost double of those of the modified models. These powers were calculated for operation frequency of 50 MHz, which is the frequency of on-board oscillator. The total energy consumption per spike (*E*_*ps*_) could be calculated as follows:


(11) $$\begin{array}{c} E_{ps}=P_{d}T_{s} \end{array}$$

where *P*_*d*_ is dynamic power and *T*_*s*_ is total time to generate an spike.

**Figure 14:**
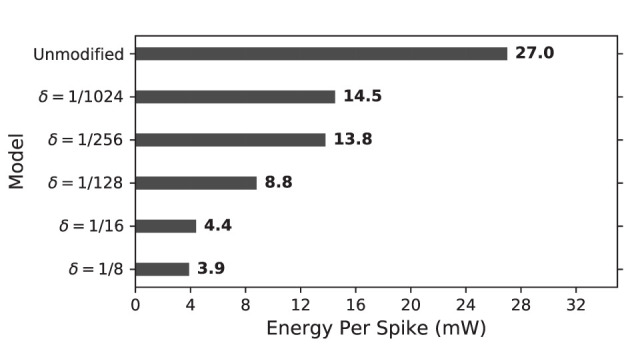
Energy consumption per spike for on-FPGA unmodified and proposed duplex Izhikevich neuron on the basis of δ. The proposed modified neuron consumes considerably lower amount of energy due to its lower switching activity.

*E*_*ps*_ for unmodified and proposed duplex Izhikevich models with different values of δ is presented in Figure 14. As results indicate, proposed models considerably reduce energy consumption per spike. This is because proposed modification not only decreases switching activity of the circuit but it also reduces total computing time. This applies to the trained network as well. As shown in Figure 12, the network with δ = 1/8 mv can process patterns almost 2.5 times faster, which also results in less switching and energy consumption as well.

#### 4.3.3 Impact of input current

To calculate the total number of clocks required to generate a spike and energy consumption measurements, it was assumed that input current of the neuron is 16 mA. Changing the input current will affect the results that presented in the previous section. As discussed in the previous sections, increasing input current results in a higher spike rate and decreasing ratio of quasi-static state period to the total period of a spike. This, in turn, leads to a lower speed up percentage.

**Figure 15:**
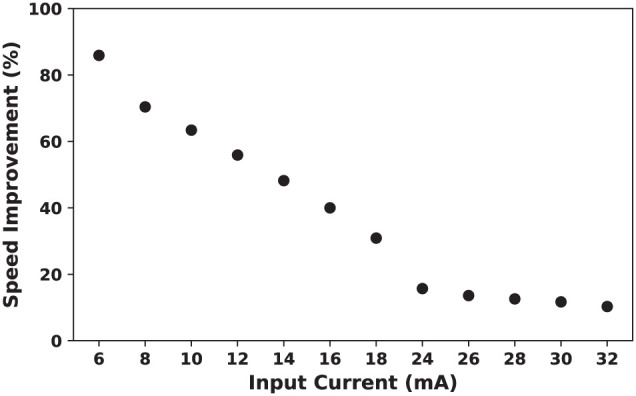
Speed up percentage for on-FPGA proposed duplex Izhikevich neuron with δ = 1/128 mv as a function of input current. The hardware for the modified model is faster for smaller values of the input current. By increasing the input current, the rate of which the speed up percentage declines becomes slower. This demonstrate that the proposed modification is most useful when neuronal activity is low.

Figure 15 shows on-FPGA speed up percentage (Equation 10) for the duplex neurons as a function of the input current. As this figure demonstrates, SUP is considerably higher for smaller input currents. This denotes that the proposed modification is most useful when neuronal activity is low.

## 5 Conclusion

In this study, a novel modification to neuron differential equations was presented to avoid unnecessary computation while simulating neurons on computers or implementing them on hardware. The proposed method is inspired by biology and benefits from the fact that most biological neurons are either silent or fire with a very slow rate. First, the proposed models were simulated for validation both as a single neuron and as a part of a network of neurons trained using STDP. Second, the impact of the proposed modification was studied on computer simulation performance in terms of the total time required to simulate neurons. The results show that the proposed modification can avoid unnecessary computations from 20 to 90 % and speed up simulation time from 2 to 18 % depending on the value of δ, input current, and time step. Furthermore, hardware was designed, described in VHDL, and simulated and implemented on FPGA. Implementation results indicated that the proposed modification accelerates the speed of hardware up to 70 %. The results for energy consumption also revealed that the proposed models can reduce energy consumption per spike from 50 % to 1/7th of its value for an unmodified neuron.

## Data availability statement

The original contributions presented in the study are included in the article/supplementary material, further inquiries can be directed to the corresponding author.

## Author contributions

MH: Writing – original draft, Writing – review & editing. AA: Conceptualization, Supervision, Validation, Writing – review & editing. MA: Conceptualization, Supervision, Validation, Writing – review & editing.
